# The population of Saudi Arabia's willingness to pay for improved level of access to healthcare services: A contingent valuation study

**DOI:** 10.1002/hsr2.577

**Published:** 2022-04-14

**Authors:** Salem Al Mustanyir, Brian Turner, Mark Mulcahy

**Affiliations:** ^1^ Department of Accounting and Finance, Cork University Business School University College Cork Cork Ireland; ^2^ Department of Economics, Cork University Business School University College Cork Cork Ireland

**Keywords:** Saudi healthcare system, health expenditure, healthcare eligibility, willingness to pay

## Abstract

**Background and aims:**

The Saudi Ministry of Health budget has surged since 2006 to put a strain on government finances at a time when the economy slowed as a result of plummeting oil prices. This study investigated the population of Saudi Arabia's willingness to pay for the healthcare services that are currently provided for free by the Saudi Ministry of Health, in return for improving their level of access.

**Methods:**

Questionnaires were used to collect data from 600 individuals in the Riyadh region. The data were elicited using payment scale format and a two‐part model was employed for data analyses.

**Results:**

The empirical analyses showed that the majority of the sample were willing to pay and found nine factors influenced people's willingness to pay—age, gender, education, employment status, nationality, marital status, current eligibility for healthcare services, possession of private health insurance, and having a chronic disease.

**Conclusion:**

The results of this study suggest that policymakers in Saudi Arabia could reduce the burden on the Ministry of Health budget, while enabling people to improve their access to healthcare services. They might be of use to policymakers to help with fund allocation and priority setting.

## 1 BACKGROUND

### 1.1 Introduction

The Saudi economy is one of the top 20 economies in the world, supported by its leading petrochemical position that finances more than two‐thirds of the government budget, and accounts for nearly a quarter of the Saudi Gross Domestic Product.^1^ However, the country ran a large budget deficit in the last decade due to the sharp and sustained decrease in oil prices, which plummeted from $110 per barrel in 2014 to a low of $22 per barrel at the beginning of 2016 (although it has since fluctuated in prices between $50 and $70 on average^2^). This decrease caused economic growth to slow from 3.6% in 2014 to −0.86% in 2017.^3^, ^4^ These indicators prompted the Saudi government to devise a new strategy—Vision 2030—to bolster its nonoil economy through various reforms, such as introducing value added tax, increasing the Ministry of Interior services fees, cutting subsidies on fuel, electricity, water and sanitation, and taxing idle lands.^5^ Meanwhile, the Saudi Ministry of Health (MOH) budget increased more than fourfold from $5 billion in 2006 to $21.8 billion in 2020.^6^, ^7^ Specifically in 2016, the MOH budget grew by 28.6%, the highest increase in the preceding 15‐year period, even though the overall government budget decreased by 2.3% that year.^8^ This increase in the MOH budget yielded the largest allocation percentage the MOH had received from the government since 2006 (11.8%), given the low level of oil prices that year. These indicators are undesirable given the government's parlous fiscal situation. Therefore, as the MOH provides healthcare services for free at the point of use and conscious of the Vision 2030 strategy, this study examines the feasibility of shifting part of the MOH healthcare costs to be incurred by end users. This would reduce the fiscal burden on the government and ensure the sustainability of the MOH healthcare services. To serve the purpose of this study, a question must be answered, which is: “What is the maximum value that the population of Saudi Arabia (SA) are willing to pay for the healthcare services that are provided by the Saudi MOH according to their current level of access?”

### 1.2 The Saudi healthcare system

#### 1.2.1 System financing

The Saudi healthcare system provides every Saudi and short‐ or long‐term non‐Saudi resident with free access to healthcare services at the point of use via three provisions as follows: (i) MOH healthcare facilities, which offers free access to all Saudis and the publicly employed non‐Saudis (and their dependents) who do not have access to their own agency's healthcare facilities.^9^, ^10^ (ii) Private employers, who must provide private health insurance (PHI) to all their employees (Saudis and non‐Saudis) and their dependents, to cover their healthcare costs in the private sector facilities, unless they work in private healthcare facility that is capable to deliver healthcare services.^10^, ^11^ (iii) Security, Defence, Universities, ARAMCO (the Saudi‐owned leading global oil company), research centers, and royal commission for Jubail and Yanbu (SDU) provides healthcare services to all their employees and their dependents^12^, ^13^, ^14^ (see Table S1 in the Supporting Information for details of limitations and nuanced eligibilities).

#### 1.2.2 Types of eligibilities in the system

**Figure 1:**
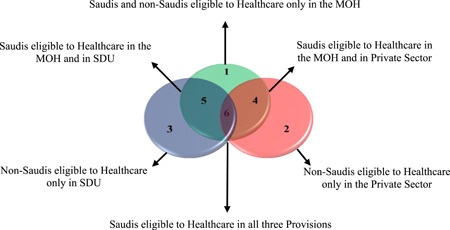
The eligibilities to the three provisions of healthcare in Saudi Arabia. *Source*: Authors' analysis of data from the Saudi Ministry of Health, year statistical book, 2017.

Such healthcare system offers six types of eligibilities as follows (see Figure 1): only in the MOH (Slice 1), only in the private sector (Slice 2), only in SDU (Slice 3), MOH and the private sector (Slice 4; i.e., when a Saudi works in the private sector), MOH and SDU (Slice 5; i.e., if a Saudi works in SDU), and all three provisions (Slice 6; i.e., when a Saudi works in SDU and is married to a Saudi working in the the private sector). Slices 2–6 comprise Part A and B. Part A contains the major eligible people who are entitled to healthcare access in their own right (for being employed publicly or privately). Part B contains the dependents, defined as those eligible to free access to healthcare, because they are dependent on someone in Part A. Slice 1 is slightly different, because all Saudis in this slice are major eligible, regardless of whether they are employed or not. On the other hand, non‐Saudis in this slice are categorized into Part A and B as the other slices. (A seventh slice could exist if a non‐Saudi works in SDU and is married to a non‐Saudi who works in the private sector. In such a case, both will be entitled to access in these two provisions. However, due to the low number of non‐Saudis working in SDU, no respondents in this study came from this slice.)

For the reason that people in Slices 4, 5, and 6 are entitled to double and, in some cases, to triple access to healthcare services, the MOH incurs extra costs and potential overuse of healthcare services, which ends up leading to longer waiting times to access necessary healthcare services in the MOH at all levels.^15^ As a result, the access of people in Slice 1 is reduced. Therefore, an examination of alternative funding models for the Saudi healthcare system was conducted.^16^ This examined the main funding options used in other economies and which would be appropriate in the Saudi context. It also proposed two reforms to take pressure off the MOH budget: (i) limiting the MOH healthcare services only to those in Slice 1, who would then have to pay compulsorily a contribution to the MOH, as their level of access will improve, because some people in Slices 4, 5, and 6 might see their access outside the MOH as sufficient. As a result, the MOH will attain funds and face reduced costs. (ii) If people in Slices 4, 5, and 6 want to maintain their access in the MOH, and those in Slices 2 and 3 want to obtain access to the MOH, then they would have to pay too, and this will also provide a source of funds to the MOH.

## 2 METHODS

### 2.1 Study sample, area, and setting

The sampling was conducted in Riyadh region, the capital of SA, and this was due to the large land mass and population living there, to ensure a reliable representation of the other regions in SA.^17^, ^18^ From six provinces of Riyadh region, 600 individuals were randomly invited from public places to share thoughts about their willingness to pay (WTP) in return for improving their level of access to healthcare in the MOH. The study took place in Riyadh city, Ad Diriyah 50 km from the city of Riyadh, Al Uyaynah 69 km, Al Muzahimiyah 102 km, Al Kharj 132 km, and Ad Dilam 145 km. Such areas involved all population classifications in terms of education, life expectancy, income, health status, employment, nationalities, and traditions, where any city in SA would have matching features. Also, the available healthcare facilities in each province differs, as the residents of some of them have full access to all types of healthcare facilities and others do not, where they need to make short or long trips to attain access to some of the advanced healthcare facilities. Eligibility was restricted to adults over the age of 18 years. The sampling was held between May and June 2018. It was felt that a sample size of 600 would be appropriate to balance a reasonable amount of statistical confidence with the feasibility of data collection.

### 2.2 Data collection

This study employed the WTP methodology, which is one of the Contingent Valuation Method techniques, as it is a widely used method to understand people's perception about participation in funding public healthcare services.^19^ The payment scale was used as an elicitation format due to its wide use in the healthcare field and its high representation of real life situations, especially in this case, where the Saudi population have a limited experience with sharing public services' costs.^20^, ^21^ Payment scale offers participants a range of values from which to choose the maximum amount that they are willing to pay. The range provided in this format eliminates the risk of the starting point bias that exists in the bidding game format and helps participants to determine their WTP to avoid potential hasty valuations that are impeded in the open‐ended format.^20^, ^22^ Payment scale can overcome range bias occurring when values in the range can influence the participants' WTP by including an open‐ended option at the end of the range.^23^, ^24^, ^25^ It can also deal with midpoint bias that arises when a participant reveals their maximum WTP by the mid value of the range by making no mid choice.^24^

To determine the scale values, the contribution percentages in some countries with more established personal contributions within their healthcare systems were tracked back. The investigation indicated that many countries started with low percentages. For example, the contribution percentage to Medibank in Australia was 2.5% in 1975,^26^ 1.45% in the United States to Medicare in 1965,^27^ Social Health Insurance in Germany and France was 6.35% and 6.8%, respectively, by employees in 1992,^28^, ^29^ Medical Savings Accounts in Singapore was between 6% and 8% in 1984,^30^ and the PHI standard plan monthly premium was 2.48% of the average monthly income in the Netherlands.^31^

Setting the payment scale took several stages. (i) Since the reforms that were introduced to meet the Saudi economic challenges have been contentious for the Saudi population, the scale was designed at 5% maximum level of contribution. (ii) To satisfy all possible choices, the values were designed in a series of ranges (i.e., 3% or more and less than 4%) as some people's maximum WTP might lay within the decimals of the percentages (e.g., 3.6%). (iii) So as not to bias the scale by restricting participants to choose percentages over zero, a zero choice was added to the scale. (iv) The scale included six positive values to eliminate the risk of midpoint bias. (v) The last option meant to be for those who have maximum WTP exceeding 5% and this is to eliminate the range bias.

This study employed a cross‐sectional questionnaire, in Arabic and English, as a data collection instrument to be filled in face‐to‐face to ensure more reliable answers. The structure of the questionnaires was guided by what had been used in WTP studies and also benefited from the guidelines that were developed by O'Brien and Gafni.^32^ The questionnaire contained four sections, the first of which provided scientific facts about the Saudi economy, and introduced the main objective of the survey. The second section was for collecting demographic and socioeconomic data of participants. The third involved three questions to identify participants' eligibilities to healthcare (participant slice). The last section contained six questions investigating people's WTP. In this section, participants were guided to answer only one question based on their answers to the third section (see Table 1).

**Table 1:** The question designated to each participants

Slice	Benefit, to (*)	Those with income	Those without income
1	*Increase	What range of percentage of your total income are you willing to pay to (*) your access to the healthcare services that are provided by the Ministry of Health?	What range of percentage of the total income of the person on whom you are dependent or from any income you might receive in the future are you willing to pay to (*) your access to the healthcare services that are provided by the Ministry of Health?
2	*Obtain		
3	*Obtain		
4	*Maintain		
5	*Maintain		
6	*Maintain		

### 2.3 Ethical standard

All procedures performed in this study involving human participation were in accordance with the ethical standards of the Social Research Ethics Committee of University College Cork, which reviewed the study materials and gave the final approval to conduct the sampling.

Informed consent was obtained from all participants in this study.

### 2.4 Data analysis

Study data were analyzed using STATA software version 15.1. Descriptive analysis was used to analyze participants' demographics and socioeconomic characteristics, percentages of WTP, and the maximum level they were willing to pay. There was no missing data in the sample. Significance of coefficients was tested using two‐sided *t* tests and results are indicated for coefficients that were significant at the 1%, 5%, and 10% levels. Moreover, the average WTP was calculated based on the mean for grouped data, assuming that 8% is the highest value for the last range in the payment scale, and this was based on the highest initial contribution percentage among the investigated healthcare systems, which was in Singapore. In addition, a two‐part model was employed on the study data to identify the factors that affect participants' decision to pay. The first used probit regression, where the marginal effects at the mean (MEM) were also used to interpret the results of the participation part. An ordinary least squares (OLS) regression was applied on the second part to find the factors that are associated with participants' WTP. However, as the OLS is focusing on the positive values, this part might include selection bias.^33^ Therefore, the inverse mills ratio was employed, which is generated using the probit coefficient and included in the OLS regression to control for selection bias.^34^ Also, as the last option that was used in the payment scale was an open‐ended option (5<), an ordered probit regression was also performed on the consumption part, which gave broadly similar results of those shown by the OLS regression. More detailed information about the methods is available in the Supporting Information.

#### 2.4.1 Demographic and socioeconomic characteristics

Table 2 shows that 74% of the study sample were males. This is attributed to traditional and religious reasons, where direct contact with female is more difficult than with male and must be carried out with extreme caution. Moreover, the figures imply that 88% of the sample were aged between 18 and 45 years. This was higher than the percentage of people in this age group in Riyadh (73%) and at the national level (72%).^35^ However, this was not unexpected as the survey was held in public places where people in these age groups are more likely to congregate than people in older age groups.^36^ The data also shows that 72% of the sample were Saudis, 56% married and 40% single. In addition, 92% have no chronic diseases, 58% were in excellent and 31% in very good self‐rated health. The data also show that 18% of participants held one of the first level of education (primary, secondary, or high school), 72% were undergraduate educated (diploma or bachelors degree), and 10% postgraduate educated (higher diploma, masters, or PhD). In addition, 88% of the study sample were employed, about 53% have PHI, and 67% of participants receive income above the Saudi average monthly income ($1700).^37^ Moreover, the data indicates that 79% of the study sample are eligible to receive healthcare in the MOH, more than half and more than third the study sample are entitled to healthcare in the private sector and SDU, respectively. It also shows that 27% of the study sample lay in Slice 1, 21% in Slice 2, <1% in Slice 3, 15% in Slice 4, 20% in Slice 5, and 16% in Slice 6.

**Table 2:** Descriptive statistics and WTP

Characteristics	Category	Participants	Participants (%)	WTP	WTP (%)	> 0 and <1	>1 and <2	>2 and <3	>3 and <4	>4 and <5	≥5	Average
Eligibility	Increase	162	27	100	61.7	36	20	11	8	14	11	2.38
	Maintain	128	21.3	83	64.8	6	21	19	15	8	14	2.98
	Obtain	310	51.7	252	81.3	39	79	45	13	19	57	3.15
	Overall	600	100	435	72.5	81	120	75	36	41	82	2.87
	Male	443	73.8	329	74.3	53	87	55	32	35	67	3.03
	Female	157	26.2	106	67.5	28	33	20	4	6	15	2.37
Age (years)	18–25	126	21	101	80.2	17	33	16	3	10	22	2.93
	26–35	273	45.5	182	66.7	33	52	36	15	13	33	2.80
	36–45	131	21.8	96	73.3	18	19	17	11	13	18	3.06
	46–55	54	9	43	79.6	10	14	3	5	3	8	2.71
	56 and above	16	2.7	13	81.3	3	2	3	2	2	1	2.65
Nationality	Saudi	434	72.3	339	78.1	64	98	56	21	32	68	2.88
	Non‐Saudi	166	27.7	96	57.8	17	22	19	15	9	14	2.84
Marital status	Single	238	39.7	171	71.8	34	51	33	11	15	27	2.67
	Married	337	56.2	245	72.7	43	63	40	23	21	55	3.05
	Divorced and widowed	25	4.2	19	76	4	6	2	2	5	0	2.39
Chronic	With	48	8	43	89.6	8	12	5	4	6	8	2.96
Disease	Without	552	92	392	71	73	108	70	32	35	74	2.86
Health status	Excellent	346	57.7	238	68.8	42	77	37	13	22	47	2.85
	Very good	186	31	148	79.6	30	30	31	14	16	27	2.93
	Good	58	9.7	42	72.4	9	10	6	8	3	6	2.73
	Fair and poor	10	1.7	7	70	0	3	1	1	0	2	3.35
Education	First level	109	18.2	73	67	18	16	17	4	10	8	2.55
	Undergraduate	433	72.2	327	75.5	55	94	52	29	26	60	2.87
	Postgraduate	58	9.7	35	60.3	8	10	6	3	5	14	3.43
Income	Below average	200	33.3	133	66.5	22	37	34	10	13	17	2.67
	Above average	400	66.7	302	75.5	59	83	41	26	28	65	2.96
Employment	Employed	527	87.8	381	72.3	67	106	63	35	36	74	2.92
	Unemployed	73	12.2	54	73.9	14	14	12	1	5	8	2.52
PHI	With	315	52.5	234	74.3	32	68	47	23	21	43	2.95
	Without	285	47.5	201	70.5	49	52	28	13	20	39	2.79

Abbreviations: PHI, private health insurance; WTP, willingness to pay.

#### 2.4.2 WTP

Table 2 indicates that 73% of the study sample were willing to pay, to improve their level of access in the MOH. It also implies that 62% of group increase, 81% of group maintain, and 56% of group obtain were willing to pay. In addition, a majority those willing to pay was found among all demographic and socioeconomic categories, with a minimum WTP of 58%. In terms of values, the data show that of those WTP, 28% were willing to pay >1% and <2%, which is the highest percentage, 19% were willing to pay >5%, and the same percentage were willing to pay >0 and <1% (see Table 2). The data analysis found that the average WTP among all the demographic and socioeconomic characteristics ranged between 2.4% and 3.4%, and the overall average was 2.9%. It was also found that the average of those in group increase was 2.4%, 3.0% for those in group maintain, and 3.1% for those in group obtain.

#### 2.4.3 Econometric analyses results

The probit and OLS regressions results in Table 3 indicate that nationality significantly influences people's decision to pay (*p* = 0.00) and also the amount that people are willing to pay (*p* = 0.02), with 48% MEM implying that Saudis are more likely to participate and 3.42 coefficient explaining that they are also willing to pay more than non‐Saudis. Participants' education was found highly associated with people's decision (undergraduate *p* = 0.01, postgraduate *p* = 0.01) and the level that people are willing to pay (undergraduate *p* = 0.03, postgraduate *p* = 0.00). The MEM of 14.6% for a participant holding an undergraduate degree and 19.7% for a participant holding a postgraduate degree indicates that they are more likely to participate relative to a participant holding one of the first level of education degrees and 0.92 coefficient that the former and 1.61 that the latter are also willing to pay more compared with those holding one of the first level of education degrees.

**Table 3:** Probit and OLS regression's results for the WTP

Independent variable	Observation	Probit *P*–*V*	Coefficient	MEM	OLS *P*–*V*	Coefficient
	Base category (female) Male	0.70	−0.06	−0.01	0.07*	0.41
Age (years)	Base category (18–25)					
	26–35	0.03**	−0.37	−0.11	0.05**	−0.81
	36–45	0.61	−0.12	−0.03	0.30	−0.37
	46–55	0.93	0.02	0.00	0.14	−0.62
	56 and above	0.82	0.1	0.02	0.79	−0.16
Nationality	Base category (non‐Saudis)					
	Saudis	**0.00** ***	**1.38**	**0.48**	**0.02** **	**3.42**
Marital Status	Base category (single)					
	Married	0.90	0.01	0.00	0.09*	0.37
	Divorced and widowed	0.38	0.32	0.09	0.17	0.76
Education	Base category (first level)					
	Undergraduate	0.01***	0.42	0.14	0.03**	0.92
	Postgraduate	0.01***	0.6	0.19	0.00***	1.61
Employment	Base category (unemployed)					
	Employed	0.23	0.3	0.10	0.07*	0.76
Income	Base category (below average)					
	Above average	0.74	−0.05	−0.01	0.59	−0.13
PHI	Base category (without PHI)					
	With PHI	0.19	−0.25	−0.07	0.00***	−0.93
Chronic Disease	Base category (without)					
	With	0.00***	0.79	0.19	0.10*	1.02
Health Status	Base category (fair and poor)					
	Good	0.76	0.14	0.03	0.83	0.15
	Very good	0.26	0.52	0.16	0.34	0.78
	Excellent	0.85	0.08	0.05	0.88	0.10
Eligibility	Base category (increase)					
	Maintain	0.01***	0.45	0.16	0.00***	1.23
	Obtain	0.00***	1.31	0.36	0.00***	4.24
Cons		0.01	−1.38	–	0.24	−3.55
IMR		–	–	–	0.16	2.79

Abbreviations: IMR, inverse mills ratio; MEM, marginal effects at the mean; PHI, private health insurance; WTP, willingness to pay.

*** Significance at 1% level.

** Significance at 5% level.

* Significance at 10% level.

In addition, study results show that chronic diseases influence participants' decision and the value that people are willing to pay (*p* = 0.00 and 0.10, respectively). The MEM interprets that a participant with chronic disease is 19% more likely to participate than those without and the coefficient (1.02) states that those who have chronic diseases are willing to pay more than those who do not. The results also showed that people aged between 26 and 35 years are less likely to participate (*p* = 0.03 and MEM −11.9%) and willing to pay less than those aged between 18 and 25 years (*p* = 0.05 and coefficient −0.81). Moreover, those in group maintain are more likely to participate (*p* = 0.01 and MEM 16.8%) and willing to pay more (*p* = 0.00 and coefficient 1.23) than those in group increase, and those in group obtain are more likely to participate (*p* = 0.00 and MEM 36.7%) compared with those in group increase, and are willing to pay the highest comparing to other groups (*p* = 0.00 and coefficient 4.24). In addition, it was found that males are more willing to pay than females (*p* = 0.07 and coefficient 0.41), employed compared to unemployed (*p* = 0.07 and coefficient 0.76), married relative to single (*p* = 0.09 and coefficient 0.37), and those with PHI are willing to pay less than those who do not have it (*p* = 0.00 and coefficient −0.93).

## 3 DISCUSSION

The data in this study shows that nearly three quarters of the participants were willing to contribute to raise a fund for the MOH to improve their level of access. Also, the figures indicate that those in Slice 2 showed a high need to obtain access and those in Slice 4 to maintain their access in the MOH, and this suggests that the healthcare services that are provided by the MOH were perceived as being better than those which are provided in the private sector. Moreover, due to the fact that the distribution of SDU healthcare facilities is centralized in the big regions and not as vast as the MOH (e.g., in Najran region, in the south of SA, the MOH provides their healthcare services for nearly 600,000 people throughout about 100 healthcare facilities, whereas there are only 3 SDU facilities there^8^, ^38^), those who are eligible to healthcare in Slices 5 and 6 reported the highest WTP percentage.

Moreover, the high level of WTP reported in this study could also be attributed to the royal grant that was introduced to offset rising costs of living in SA. The stated grant, which started at the end of January 2018, includes $267 monthly payments to be given to the civil and military personnel, and at a lower level to the public and private pensioners, as well as social security beneficiaries and students.^39^ In addition, hundreds of the middle and large size private companies have cooperated and followed the royal decree.^40^ In fact, every 1% in the designed scale in this study equates to about $17 per month for someone on the average income in SA. This amount ($17) accounts for small proportion of the royal grant and is very low compared with the size of benefit that will be guaranteed from the MOH. Therefore, this was perceived highly attractive for many categories of people.

On the other hand, if the publicly and private employees and pensioners contributed the average WTP (2.9%), the MOH would be able to reduce its fiscal reliance on the government budget by 38.8% (14.3 million employees and pensioners in 2019 × $1,700 the average monthly income in 2019 × 12 months × 2.9% contribution ÷ $21.8 Billion the MOH 2020 budget).^37^, ^41^, ^42^ More funds could possibly be attained from the majority of the population (other than employees and pensioners), which represent 58% of the total population, and there is also the possibility that the MOH will experience reduced costs as some people may perceive their access outside the MOH as sufficient, thereby reducing the volume of treatments.

Study results showed that non‐Saudis were less willing to participate and were willing to pay less than Saudis, which may be due to the fact that the majority of non‐Saudis receive income below the Saudi monthly average income ($1700). Specifically, 88% of non‐Saudis who work in the private sector (73% of total non‐Saudis in SA) are in receipt of income <$800 per month.^43^ In addition, their dependents are in receipt of no benefit from the government. Therefore, non‐Saudi employees tended to save rather than spending to attain better quality of healthcare and their dependents were also not willing to pay so as not to put more burden on those on whom they are dependent. Consequently, their WTP was lower than Saudis.

In addition, the findings of this study confirm the previous studies related to education, whereby when people's education increases, the decision to pay and the level of contribution were more likely to increase.^44^, ^45^ Moreover, it was found that unemployed people were willing to pay less relative to employed, because the former would be in receipt of no income or could be entitled to a low compensation from the Saudi government; therefore, they valued the money more than the employed people. The same is true for the females who were less widely employed in SA; therefore, they tended to pay less than males (employed females in SA represent <17% of total jobs in 2018).^46^

In terms of chronic diseases, the study result seems logical, as people with specific illnesses would be more careful to look after their health. Paradoxically, and perhaps surprisingly, the findings of the literature review suggest that healthy people were more willing to pay than the less healthy.^47^, ^48^ In terms of PHI, the data indicated that people who have PHI were willing to pay less than those who do not. In fact, the data showed that the majority of both categories were willing to pay a percentage over the overall average (with PHI 2.9% and without 2.8%). However, when it comes to the high percentages, it was found that those without PHI tended to place higher values than those with PHI (see Table 2).

The findings of the study came in line with previous studies in respect of the negative effect of age at WTP.^48^, ^49^, ^50^ where >25% of those aged between 18 and 25 years were willing to pay values higher than 4% compared with 17% of those aged between 26 and 35 years (see Table 2). Willingness to pay was lower for older age groups (ages 46–55 and 56 years and above). The latter might be related to the fact that people in the oldest age category are more likely to be in receipt of pension income, which would be lower than the earnings of younger age groups who are more likely to be in employment. An alternative explanation might be that they are more accustomed to being able to access services for free and might be less inclined to change this. However, the sample size of those aged 56 years and older is also quite small, so caution would be advised in interpreting this finding.

In addition, the figures indicated that 29% of married participants were willing to pay more than 3% compared with 22% of singles. Furthermore, the findings of this study indicate that people in group maintain and obtain were more willing to participate and to pay than people in group increase, where people in group obtain were the most willing. This shows how valuable the healthcare services that are provided by the MOH are to groups maintain and obtain, given the fact the latter receive the lowest income compared to the two other groups.

## 4 CONCLUSION

This study examined the WTP among a population with limited experience of funding public services, and in a unique healthcare system that provides healthcare services to the entire population for free at the point of use through three different provisions. The government in this system funds the MOH services at no cost to the Saudi citizens and non‐Saudis who work for the government. The same is applicable for those eligible to SDU and those eligible to the private sector do not have to pay significant deductibles or copayments.

In addition, the findings of this study have generated an important policy contribution to the Saudi government especially at this critical time, due to the economic challenges and the need to diversify the public financial resources.

In conclusion, this study suggests that involving the Saudi population to fund their healthcare in the MOH and restricting the MOH services only to those in Slice 1 and who are willing to pay from other slices, is a feasible plan for the Saudi MOH to help in ensuring the sustainability of public healthcare services. In addition, the access suspension of those in group maintain, will reduce the MOH budget and take pressure off its facilities, thereby improving access for those in Slice 1 and who want to maintain or obtain access from other slices.

### 4.1 Study limitation

The sample was selected from Riyadh region and this might raise the question of the validity of extrapolating the results to the entire country. Therefore, the current study tried to minimize the effect of such limitations by selecting the sample from six provinces from different distances to where most of the healthcare facilities exist to ensure population diversity.

## AUTHOR CONTRIBUTIONS

**Salem Al Mustanyir**: Conceptualization; data curation; formal analysis; investigation; methodology; project administration; resources; software; visualization; writing—original draft. **Brian Turner** and **Mark Mulcahy**: supervision; validation; writing—review and editing. All authors have read and approved the final version of the manuscript. Salem Al Mustanyir had full access to all of the data in this study and takes complete responsibility for the integrity of the data and the accuracy of the data analysis.

## CONFLICTS OF INTEREST

The authors declare no conflicts of interest.

## Supporting information

Supporting Information

## Data Availability

All data have been included in this manuscript and any additional data can be provided up on request.
