# Supplementary material for: The population of Saudi Arabia's willingness to pay for improved level of access to healthcare services: A contingent valuation study
Source: Health Sci Rep. 2022 Apr 14;5(3):e577. doi: 10.1002/hsr2.577 (PMC9059215; doi:10.1002/hsr2.577)
Supplement: Supplementary file 1 — Supporting Information [file HSR2-5-e577-s001.docx]

**The population of Saudi Arabia’s willingness to pay for improved level of access to healthcare services: a contingent valuation study**

**International Journal of Health Planning and Management**

Salem Al Mustanyir ^1^, Brian Turner ^2^, Mark Mulcahy ^3^

^1^ Corresponding author, Department of Accounting and Finance, Cork University Business School, University College Cork, Cork, Ireland. E-mail: [Salmustanyir@hotmail.com](mailto:Salmustanyir@hotmail.com) Telephone Number: 00966568688292. ORCID: 0000-0002-2853-1730

^2^ Department of Economics, Cork University Business School, University College Cork, Cork, Ireland. ORCID: 0000-0003-2545-8336

^3^ Department of Accounting and Finance, Cork University Business School, University College Cork, Cork, Ireland. ORCID: 0000-0002-1201-646X

**Details of limitations and nuanced eligibilities**

The main aspects of eligibility for healthcare services in Saudi Arabia are contained in Section 1.2 of the paper. However, there are some nuances relating to these, which are worth noting for the sake of completeness (see Table 1).

**Table 1 Limitations and Additional Eligibilities to the Saudi Healthcare System**

| Provision | Limitation |
| --- | --- |
| In all Provisions | Visitors are obliged to obtain private health insurance before going to SA. |
| The first provision (MOH) | *Free access is also applicable to some limited non-Saudi categories, such as the Gulf Cooperation Council (GCC) citizens, Ministry of Defence non-Saudi employees, non-Saudis with single sponsorship (i.e. private housekeepers, personal drivers, nursemaids, and farmers in Saudi households), the disabled, orphans, non-Saudis with specific illness (i.e. AIDS, Hepatitis, Leprosy, and Tuberculosis), minorities of displaced tribes, prisoners, pilgrims, students with sponsorship, Saudi wives, sons and daughters of a Saudi mother, and mothers to a Saudi.  *If the public agency provides healthcare services through its own facilities to its employees and their dependents such as the security, universities, ARAMCO, and research centres, then the non-Saudi employed in this sector cannot freely access MOH healthcare facilities. |
| The third provision (SDU) | All people who work in SDU are fully covered in the related healthcare facilities. (e.g. people who work in the Ministry of the Interior (MOI) and their dependents get free access to all MOI healthcare facilities). The same is true for the other SDU agencies, such as the Ministry of National Guards (MONG). However, if someone is eligible to healthcare in one, they cannot access the other (e.g. the MOI employees cannot access healthcare in the MONG). |

**The results of the ordered probit regression on the consumption part**

For the reason that the last option that was used in the payment scale was an open-ended option (5 <), this option could be categorical rather than continuous. Given the presence of uncertainty in the nature of the order of the payment scale used in this study, it was necessary to perform a robustness test on the participation part figures to investigate the degree to which a different model would function correctly and give the same significant results as the OLS that was used initially, and also how these estimates to the payment scale (that the last option is continuous or categorical) could be sensitive to changing the model.

The OLS model assumes that the dependent variable is continuous (the difference between 1 and 2 is equal to the difference between 2 and 3). However, because some uncertainties arose regarding the nature of the payment scale that the last option could be categorical rather than continuous, this was addressed using the ordered probit regression, which is used specifically to deal with the variable that is categorical and ordered, which could be the case in this part of the study ^1^.

When the ordered probit model was performed on the participation part, the results showed that both models (OLS and ordered probit) are broadly similar, where most of the variables that were found significant in the OLS regression, were also found to be significant when the ordered probit model was used, with three more variables found to be significant in the OLS (those aged between 25 and 35, married participants, and those with chronic diseases), and these few additional results were due to the different assumptions of each model, which is likely to occur. Finally, the overall results are robust, and show that the majority of the variables are similarly significant in both models (see Table 2).

**Table 2 OLS and Ordered Probit Regression’s Results for the Willingness to Pay**

| **Independent Variable** | **Observation** | **OLS P-V** | **Coefficient** | **O-Probit P-V** | **Coefficient** |
| --- | --- | --- | --- | --- | --- |
| **Gender** | **Base Category (Female)** |  |  |  |  |
|  | **Male** | **0.07** | **0.41** | **0.09** | **0.23** |
| **Age** | **Base Category (18 - 25)** |  |  |  |  |
|  | **26 - 35** | **0.05** | **-0.81** | 0.11 | -0.44 |
|  | **36 - 45** | 0.30 | -0.37 | 0.47 | -0.15 |
|  | **46 - 55** | 0.14 | -0.62 | 0.23 | -0.30 |
|  | **56 & Above** | 0.79 | -0.16 | 0.94 | 0.02 |
| **Nationality** | **Base Category (Non-Saudis)** |  |  |  |  |
|  | **Saudis** | **0.02** | **3.42** | **0.00** | **2.84** |
| **Marital Status** | **Base Category (Single)** |  |  |  |  |
|  | **Married** | **0.09** | **0.37** | 0.12 | 0.20 |
|  | **Divorced & Widowed** | 0.17 | 0.76 | 0.24 | 0.38 |
| **Education** | **Base Category (First Level)** |  |  |  |  |
|  | **Undergraduate** | **0.03** | **0.92** | **0.03** | **0.62** |
|  | **Post-graduate** | **0.00** | **1.61** | **0.00** | **1.04** |
| **Employment** | **Base Category (Unemployed)** |  |  |  |  |
|  | **Employed** | **0.07** | **0.76** | **0.04** | **0.55** |
| **Income** | **Base Category** |  |  |  |  |
|  | **Above Average** | 0.59 | -0.13 | 0.26 | -0.16 |
| **PHI** | **Base Category (Without PHI)** |  |  |  |  |
|  | **With PHI** | **0.00** | **-0.93** | **0.00** | **-0.61** |
| **Chronic Disease** | **Base Category (Without)** |  |  |  |  |
|  | **With** | **0.10** | **1.02** | 0.13 | 0.62 |
| **Health Status** | **Base Category (Fair & Poor)** |  |  |  |  |
|  | **Good** | 0.83 | 0.15 | 0.94 | -0.03 |
|  | **Very Good** | 0.34 | 0.78 | 0.47 | 0.37 |
|  | **Excellent** | 0.88 | 0.10 | 0.92 | -0.03 |
| **Eligibility** | **Base Category (Increase)** |  |  |  |  |
|  | **Maintain** | **0.00** | **1.23** | **0.00** | **0.87** |
|  | **Obtain** | **0.00** | **4.24** | **0.00** | **3.48** |
| **Cons** |  | 0.24 | -3.55 | - | - |
| **IMR** |  | 0.16 | 2.79 | 0.21 | 1.71 |

**Source:** Authors’ analysis of data from the Study Survey.

**Reference**

**1.** Borooah VK. *Logit and probit: Ordered and multinomial models*: Sage; 2002.
